# Hip abductor strengthening in patients diagnosed with knee osteoarthritis – a systematic review and meta-analysis

**DOI:** 10.1186/s12891-022-05557-6

**Published:** 2022-06-29

**Authors:** Dias Tina Thomas, Shruthi R, Ashish John Prabhakar, Patel Vivekbhai Dineshbhai, Charu Eapen

**Affiliations:** Department of Physiotherapy, Kasturba Medical College, Mangalore, Manipal Academy of Higher Education, Manipal, India

**Keywords:** OA of the knee, Hip abductor resistance training, Strength training

## Abstract

**Background:**

Globally osteoarthritis of the knee is a leading cause of disability. Hip abductor strength and activation are essential for maintaining postural balance during transfers and are related to joint loading and progression during weight-bearing activities. Strength deficits in the hip abductors might cause a reduction in the lower extremity force generation, thereby causing stress on the medial tibiofemoral joint. The aim of this systematic review is to assess the effectiveness of hip abductor strengthening on knee joint loading, knee pain and functional outcome measures in patients with knee osteoarthritis.

**Methods:**

Database such as Scopus, PubMed, EMBASE, Cochrane Central Register of Controlled Trials (CENTRAL) database and PEDro were reviewed to recognize the trials published in English from inception to December 2020. Randomized controlled trials that studied the effectiveness of hip abductor strengthening in subjects with knee osteoarthritis and its impact on knee joint loading, knee pain and functional outcome measures were included. RevMan 5.4 was used for meta-analysis and forest plot construction. Quality assessment of the included studies was carried out using the PEDro scale.

**Results and discussion:**

The search yielded 260 results of which 29 full-text articles were screened. The review includes 7 randomized controlled trials and 3 studies with good methodological quality were included for meta-analysis. The meta-analysis of the articles favored hip abductor strengthening intervention over the control group. Hip abductor strengthening had significantly reduced the VAS [ SMD = -0.60[-0.88, -0.33] *p* < 0.0001]at 95% CI and improved the WOMAC scores [SMD – 0.75[-1.05,-0.45] *p* < 0.0001] at 95% CI. All of the included studies concluded that strengthening the hip abductor muscle had a positive impact on knee pain and functional outcomes.

**Conclusion:**

The current study found high-quality evidence to support the use of hip abductor muscle strengthening exercises as a rehabilitative treatment for subjects with knee osteoarthritis.

**Trial registration:**

CRD42021256251.

**Supplementary Information:**

The online version contains supplementary material available at 10.1186/s12891-022-05557-6.

## Introduction

Osteoarthritis (OA) is a degenerative, localized joint disease that affects approximately one-third of all individuals, with the prevalence of the disease increasing with age [1]. Many joints are affected by OA, including the large, weight-bearing joints (hips and knees), as well as the spine, hands, feet, and shoulders. The knee is the most commonly impacted weight-bearing joint by OA, with the condition primarily impacting the medial compartment of the tibio-femoral joint [2, 3]. We discovered that the global incidence of knee OA in people aged 20 and up was 203 per 10,000 person-years. Similarly, there will be around 867 million people (20 years and older) with incident knee OA throughout the world in 2020 [4]. In India, OA is the 2^nd^most common rheumatological problem, with a disease prevalence of 28.7% [5]. The pathology involved includes physiological and biological changes to the hyaline cartilage, surrounding bones, soft tissue, ligaments, synovial fluid and muscles associated with sclerotic alterations in the subchondral bone synovial tissue proliferation and osteophyte formation [6]. All these changes at and within the joint can cause impairments which may include joint swelling, limited range of motion, pain, decreased strength, abnormalities in gait and stiffness [6].

Compression and loading of the knee joint are reversible factors that contribute to disease progression [7]. Compressive forces on the knee caused by knee adduction moment on the medial compartment of the joint are associated with the severity of the disease and intensity of pain [8–10]. In addition, decreased strength of the quadriceps is one of the contributing factors for the onset of the disease [11]. Hence, strengthening the quadriceps muscles helps protect the knee joint cartilage by absorbing the loads placed on the joint [12–14].

It is known that the strength of the hip musculature may directly affects knee joint loading, leading to the progression of the disease [12]. During walking, there is an increase in the dynamic load on the knee. The ground reaction force travels to the medial aspect of the knee during stance, generating an external knee adduction moment, which forces the knee outwards, compressing the medial joint and stretching the lateral joint components [15, 2, 16].

Subjects with knee OA present with weak hip abductors as a result of which there is a decrease in their isokinetic strength, isometric strength and explosive force [17–21]. Hip abductor weakness of the stance limb causes a fall in the pelvis of the swing limb. As the line of gravity changes away from the stance knee, the medial joint compressive forces and knee adduction moment increase, resulting in progressive deterioration [22, 23]. Hip abductor weakness is associated with functional decline as it impacts force generation [24] thereby altering the knee joint loading and structural progression during weight-bearing movements.

Despite the existence of literature on the efficacy of various types of exercises in patients with knee osteoarthritis, to the best of our knowledge no review collectively describes the influence, effectiveness, and importance of hip abductor muscle strengthening in knee osteoarthritis. As a result, the goal of this review is to identify and examine the existing evidence on the effects of hip abductor muscle strengthening on knee pain, functional outcomes and knee joint loading in subjects with knee OA.

## Methods

This systematic review and meta – analysis was prospectively registered with PROSPERO, on 20/06/2021 bearing the registration id: CRD42021256251. According to the Preferred reporting items for systematic reviews and Meta-Analyses (PRISMA) guidelines, the review protocol and reporting of the systematic review were carried out.

### Search strategy

To find relevant articles the following five electronic engines were searched: PubMed, Scopus, Cochrane Central Register of Controlled Trials (CENTRAL) database, Physiotherapy Evidence Database (Pedro), and Experta Medica database (EMBASE) for articles that established the efficiency of hip abductor strength training for subjects with knee OA. The studies included were written in English. Two individual investigators conducted the search using a combination of two primary keywords: “Knee OA” (population) AND “strength training” with the prefix “Hip abductor” Boolean operators “AND” “OR” were used to merge the two keywords (Table 1). The search methods were changed depending on the databases. The publication dates were unrestricted, and the articles published between inception to December 2020 were included in the review.

**Table 1:** – Search strategy

*Knee osteoarthritis
Knee osteoarthritides
Osteoarthritis of knee
Osteoarthritis of the knee
*Hip abductor training
Hip abductor resistance training
Hip abductor strengthening
Hip abductor strength training
Hip abductor strengthening program
Hip abductor exercise program
Hip abductor weight-bearing strengthening program
Hip abductor weight-bearing exercises

((Hip abductor training) OR (hip abductor resistance training)) OR (hip abductor strengthening)) OR (Hip abductor strength training)) OR (Hip abductor strengthening program)) OR (Hip abductor exercise program)) OR (Hip abductor weight bearing strengthening program)) OR (hip abductor weight-bearing strengthening programs)) OR (hip abductor weight-bearing exercises)) AND ((((knee osteoarthritides) OR (knee osteoarthritis)) OR (osteoarthritis of knee)) OR (osteoarthritis of the knee))

### Eligibility criteria

Conference abstracts, case reports, observational studies and clinical commentaries were excluded. Studies generalizing hip-strengthening exercises were excluded. Articles including conditions like systemic arthritic conditions, tibial osteotomy, hip or knee joint replacement and any other muscular or disease neurological that may affect the lower extremity were eliminated.

Studies meeting the following inclusion criteria were incorporated in the review -

Population—The subjects in the study must have a diagnosis of knee OA of any age group, grade, or gender.

Intervention—Randomized control trials (RCT’s) comparing effects of hip abductor strengthening exercises with other exercises of the lower extremity or no treatment on pain, knee joint loading and functional outcomes were included.

Outcome Measures included for the systematic review were pain, quantified using Visual Analogue Scale(VAS), Numerical Pain Rating Scale (NPRS), self-reported physical function, Knee Injury and Osteoarthritis Outcome Score (KOOS), Western Ontario and McMaster Universities(WOMAC), quality of life measured using Short Form- 36 (SF-36), physical function tests and medial joint loading.

### Study selection

**Fig. 1:**
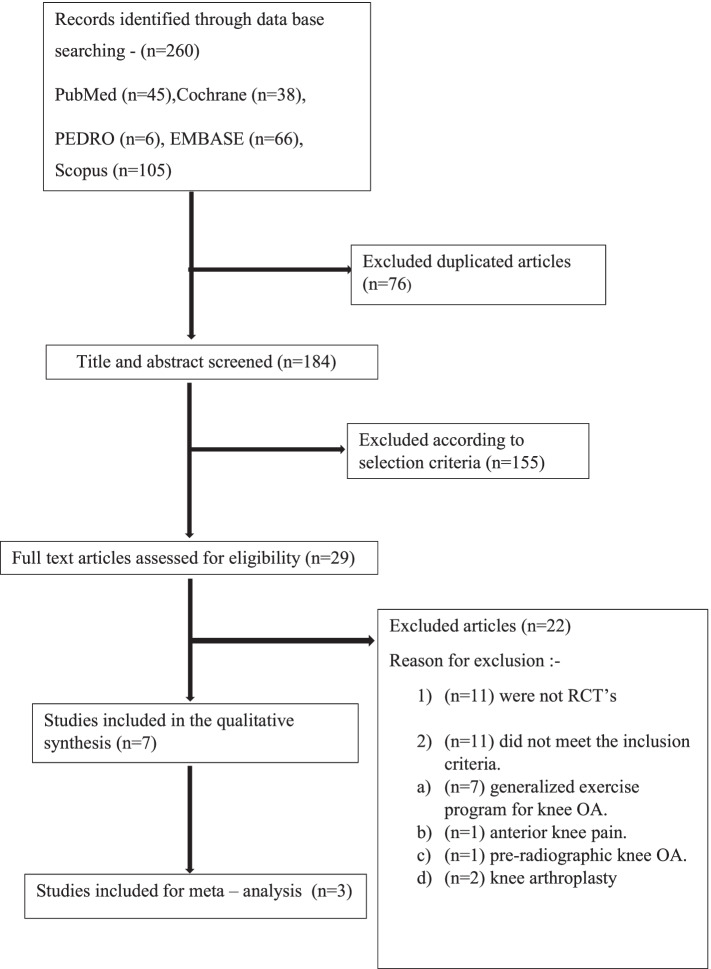
PRISMA flow chart

The search was conducted by two independent reviewers (DT, SR) on various databases, following which all the identified studies were imported into Mendeley reference manager. The titles and abstracts were screened by two independent reviewers (DT, SR) using the online software Rayyan QCRI. Ambiguities between the reviewers (DT, SR) were bought to a consensus by discussing with the third reviewer (AP). The eligibility assessment under the inclusion–exclusion criteria was carried out by reviewing full-text articles. The results of the search are presented in the Preferred Reporting Items for Systematic Reviews and Meta-analyses (PRISMA) flow diagram (Fig. 1).

### Data extraction

One reviewer (DT) obtained data from the included articles, which was then substantiated by a second reviewer (SR) and entered into a standard form developed for the review by both reviewers (DT, SR). Information about the authors, journal, year of publication, characteristics of the subjects (age, inclusion criteria, gender, sample size), method (i.e., design, subjects, intervention, measures), outcome assessed, details of the interventions (parameters, frequency, intensity, type, time) and comparison groups, and the adverse events seen during the course of treatment were noted. All studies reported pre-and post-intervention scores.

### Quality assessment

The two reviewers (DT, SR) autonomously conducted a procedural quality assessment of the studies based on the PEDro scale. Studies that scored less than 6 on the PEDro scale were not included for meta-analysis. Ambiguities among the two reviewers were resolved by the third reviewer (AP). The PEDro scale was used to gauge the quality of the included studies. The PEDro scale is the sum of 11 questions' responses. Each question is worth one point, which assesses the trials' statistical significance and internal validity.

### Data management and synthesis

In the articles that were obtained, the outcome measures were analysed. The intended result was sought, and statistical values were recorded for it. The effectiveness of hip abductor strengthening on pain and functional outcomes was calculated using the mean, standard deviation, and mean difference. The pre- and post-intervention changes in values between groups were compared and the mean difference was computed. For pre- and post-analysis, the values of secondary outcomes of interest were also recorded and compared.

**Fig. 2:**
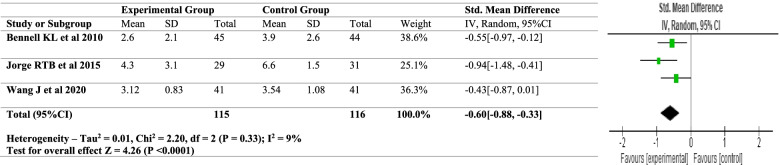
Forest plot – for VAS

**Fig. 3:**
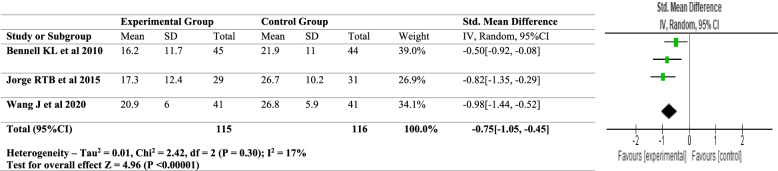
Forest plot – WOMAC

Meta-analysis was performed on the homogenous outcomes in the present study, namely pain (VAS) and functional outcome (WOMAC fucntion). The random-effects model was used for the meta-analysis since significant heterogeneity was expected among the trials. The Chi^2^ statistic was used to examine heterogeneity among the selected studies and the I^2^ statistic was used to assess heterogeneity (> 60 percent was considered substantial heterogeneity). The meta-analysis was carried out using RevMan 5.4 software. The forest plots for VAS and WOMAC function are shown in Figs. 2 and 3. A descriptive analysis based on mean differences pre- and post-intervention i.e., the follow up scores from baseline was undertaken.

## Results

After deleting duplicates 184 articles were screened from 260 results found—PubMed (*n* = 45), Cochrane (*n* = 38), PEDRO (*n* = 6), EMBASE (*n* = 66), and Scopus (*n* = 105). 155 articles did not meet the inclusion criteria during the title and abstract screening; hence the remaining 29 full-text articles were reviewed. Out of the 29 full-text articles screened, (*n* = 11) were not RCT’s and (*n* = 11) were excluded since the intervention was not hip abductor strengthening and the studies addressed other knee problems. After reviewing the full text, 7 RCT’s were chosen. On assessing the quality of the study only 5 [25–29] articles had a PEDro score ≥ 6 and 2 studies had a PEDro score (Table 2) of 5 [30] and 3 [31]. Studies with PEDro score < 6 were excluded from the meta-analysis due to low methodological quality. A total of 388 people with with knee osteoarthritis of either age, grades and gender participated in the trials. The study selection process is depicted in the PRISMA flowchart in the Fig. 1

**Table 2:** PEDRO quality scoring of the studies ✓

Trial	1	2	3	4	5	6	7	8	9	10	11	Score
Wang et al	✓	✓	✓	✓	x	x	✓	✓	x	✓	✓	7/10
Yuenyongviwat et al	✓	✓	✓	✓	x	x	x	✓	✓	✓	✓	7/10
Singh et al	✓	✓	x	✓	x	x	✓	✓	x	✓	✓	6/10
Bennell et al	✓	✓	✓	✓	x	x	✓	✓	✓	✓	✓	8/10
Jorge et al	✓	✓	✓	✓	x	x	✓	✓	✓	✓	✓	8/10
Elizabeth A. Sled	✓	x	x	✓	x	x	x	✓	✓	✓	✓	5/10
Chaudhary Ashok	✓	✓	x	✓	x	x	x	x	x	x	✓	3/10

Items for methodological quality criteria (2–11 were considered for total score):

1: Were the eligibility criteria specified?

2: Were subjects randomly allocated to groups?

3: Was allocation concealed?

4: Were groups similar at baseline for the most important prognostic indicators?

5: Were all subjects blinded?

6: Were all therapists who administered therapy blinded?

7: Were all assessors who measured at least one key outcome blinded?

8: Were measures of at least one key outcome obtained from > 85% of the subjects initially allocated to groups?

9: Did all subjects (for whom outcome measures were available) receive the treatment or control condition as allocated, or, where this was not the case, was data for a least one key outcome analyzed by intention to treat?

10: Were the results of between-group statistical comparisons reported for at least one key outcome?

11: Did the study provide both point measures and measures of variability for at least one key outcome?

### Study characteristics

The size of the samples ranged from 30 to 97 subjects. One study included exclusively female subjects [26], while others had both male and female subjects. Yuenyongviwat et al. [29] included an additional inclusion criteria for research subjects: they had to be able to walk without assistance, have a knee flexion of more than 90 degrees and have a varus of fewer than 10 degrees. Subjects with a VAS of 7 were considered in the trial by Wang et al. [28]. All the trials that were included in the study were analyzed based on American College of rheumatology criteria. All the studies used the Kellgren Lawrence score for radiological grading. Renata et al. [26] reported to have included Kellgren Lawrence grades I & II, whereas the rest of the studies included Kellgren Lawrence grading II & above.

A summary of exercises, intensity and frequency are presented in (Table 3) in detail. Wang et al. [28] and Sled et al. [30] measured the effect of hip abductor exercises alone, whereas other studies [25–27, 29, 31] measured the effect of hip abductor strengthening in comparison to quadriceps exercises or routine rehabilitation. Exercises for all the studies were given with a TheraBand or weight cuff. Apart from side-lying hip abduction, Wang et al. [28] included pelvic lift training, which required the subject to stand with one leg off the side of a 10 cm step. Initially, the leg that is lower than the step level was trained. Then, to raise the opposite leg to the same level as the step, the participant had to engage the stance leg hip abductor. Except for one home-based and supervised study [27], the exercise intervention supplied to the subjects was all supervised. The details of each study are summarized in (Table 4).

**Table 3:** Description of the intervention used

Author	Frequency (F) and duration (D)	Intensity of exercise	Type of exercise
Bennell et al	F: 5/week D: 12 weeks Supervised and home-based	With ankle cuff weights or elastic bands, 3 sets of 10 repetitions	The SG completed six exercises in side-lying and standing to strengthen the hip abductor and adductor muscles Subjects in the CG did not receive any additional treatment or do any home activities during the 12-week period
Jorge et al	F: 2/week D: 12 weeks Supervised	The 1RM was used as the starting load The regime consisted of two sets of eight repetitions, with the first set utilising 50% of 1RM and the second set utilising 70% of 1RM. Between sets, there was a one-minute rest period	SG—progressive resistance exercise programme (PRE) that included four separate hip abduction/adduction movements performed with free weights on two gym machines (knee flexion–extension and abduction and adduction) (supplementary material). A five-minute warm-up on an exercise bicycle followed the activities
Singh et al	F: 5/week D: 6 weeks supervised	Subjects began by performing 50 percent of their one-repetition maximum. After then, a new 1 RM was measured every week, and the load was gradually raised The duration of the contraction was 5–10 s, depending on the tolerance of the subjects. Between repetitions, there was a ten-second break and a one-minute break between sets	SG — Side-lying with weight cuffs and traditional exercises were used to improve the hip abductor muscles CG – received the traditional knee exercises. Static quadriceps, straight leg lift and short arc terminal extension
Yuenyongviwat et al	F: 2/week D: 10 weeks Supervised	4 sets- of 10 repetitions twice a day	SG—Hip abductor and quadriceps strengthening activities were performed. Subjects were instructed to lie down in a side-lying position and abduct the hip to a 45-degree abduction posture, which they held for 10 s. The patient's ankle was wrapped with a sandbag which was weighted per protocol while completing quadriceps workouts or hip abduction exercises Only quadriceps strengthening exercises were done by CG
Wang et al	F: 1/day D: 6 weeks Supervised	3 sets of 10 repetitions progression to a greater resistance level was possible when subjects could perform 20 repetitions	SG – hip abductor strength–based exercises CG – Quadriceps femoris strength–based exercises
Elizabeth A. Sled et al	F:1/day D: 3 to 4 times per week for 8 weeks Unsupervised – Home based	Progression to greater resistance levels occurred when participants could perform the exercise without fatigue for 20 repetitions	SG-Side-lying resistive exercises for the hip abductor muscles, progressing to using resistance bands, standing single-leg stabilization exercises, progressing to standing hip abduction using resistance bands CG-daily activities and refrain from beginning any new exercise program
Chaudhary Ashok	F:1/day D:6 days for weeks Supervised	3 sets of 10 repetitions at 10 RM performed in side lying with resistance 3 sets of 10 repetitions at moderate resistance performed in standing with band 3 sets of 10 repetitions. Performed in unipedal stance with 5 s hold	SG- hip abductor strength–based exercises CG- Short wave diathermy, stretching exercises, range of motion exercises, strengthening exercises

*CG* Control group, *SG* Strengthening group, *F* Frequency, *D* Duration, *1RM* One repetition Maximum

**Table 4:** Description of the studies and pre- post-intervention values

**STUDY**	**PARTICIPANTS **	**OUTCOMES**	**RESULTS**	**OUTCOMES**	PRE – INTERVENTION		POST – INTERVENTION	
					SG	CG	SG	CG
Bennell et al	Sample size (*n* = 89) Strengthening Group (SG) (*n* = 45) Control Group (CG) (*n* = 44) Mean age of the SG group- 64.5 (9.1) Mean age of the CG group- 64.6 (7.6) Kellgran Lawrence (KL) grade 2 and above were included.	A 3-dimensional gait analysis to identify the knee adduction moment.11-point NPRS Maximal isometric strength of hip musculature Step test and timed stair ascent and descent task	Hip abductor and adductor strengthening alone improved pain, function, and hip strength but did not alter knee adduction when compared with no treatment.	KAM VAS WOMAC function Step test Timed stair task Hip abduction torque	3.2 ± 1.0 4.3 ± 2.0 24.8 ± 10.9 16.2 ± 3.7 8.0 ± 2.7 0.9 ± 0.3	2.9 ± 0.9 4.1 ± 2.5 23.7 ± 11.8 16 ± 2.9 8.2 ± 2.3 0.8 ± 0.3	3.3 ± 0.9 2.6 ± 2.1 16.2 ± 11.7 18 ± 4.3 7.0 ± 2.2 1.0 ± 0.3	2.9 ± 0.9 3.9 ± 2.6 21.9 ± 11.0 16.9 ± 2.8 7.9 ± 1.8 0.9 ± 0.3
Jorge et al	Sample size (*n* = 60) Strengthening Group (SG) (*n* = 29) Control Group (CG) (*n* = 31) Mean age of the SG group-61.7 ± 6.4 Mean age of the CG group-59.9 ± 7.5 Kellgran Lawrence (KL) grade 1-4 were included	Pain (VAS scale) Physical Function (WOMAC) Walking Distance – 6MWT Strength – 1RM Quality of life – SF-36	Lower extremity progressive resistance exercise (inclusive of hip abductor and adductor strengthening) is effective when compared with a waitlist no treatment control group	VAS WOMAC function SF 36 6MWT	7.0 ± 1.3 27.7 ± 9.3 39.3 ± 16. 357.1 ± 56.9	7.0 .0 ± 1.2 28.4 ± 10.6 32.4 ± 16.0 330.2 ± 55.9	4.3 ± 3.1 17.3 ± 12.4 49.8 ± 21.9 69.5 ± 60.8	6.6 ± 1.5 26.7 ± 10.2 30.8 ± 16.8 343.1 ± 54.7
Singh et al	Sample size (*n* = 30) Strengthening Group (SG) (*n* = 15) Control Group (CG) (*n* = 15) Mean age of the SG group- 55.33 ± 3.99 Mean age of the CG group- 54.86 ± 4.35 Kellgran Lawrence (KL) grades 2 and 3 were included.	Hip abductor strength by modified sphygmomanometer Physical Function (WOMAC) Walking Distance – 6MWT	Hip abductor strengthening and quadriceps exercise, when compared with quadriceps exercise alone, produced superior outcomes on function and walking distance.	WOMAC function 6MWT Hip Strength	116.13 ± 16.01 280.13 ± 28.27 97.30 ± 9.42	113.93 ± 16.40 279.11 ± 49.23 95.47 ± 18.10	59.46 ± 12.44 378.37 ± 27.81 124.70 ± 9.45	82.73 ± 13.96 320.07 ± 46.40 96.56 ± 18.45
Yuenyongviwat et al	Sample size (*n* = 97) Strengthening Group (SG) (*n* = 42) Control Group (CG) (*n* = 44) Mean age of the SG group- 62.8 ± 6.80 Mean age of the CG group- 62.5 ± 8.4 Kellgran Lawrence (KL) grades 2 and 3 were included.	Knee Injury and Osteoarthritis Outcome Scores (KOOS)	All KOOS subscales were significantly improved in both groups after 10 weeks. No significant difference in the scores between either group at 2–10 weeks after treatment. Nevertheless, the effects of exercise for pain, symptoms, function in daily living and knee related quality of life were found to have faster improvement within the hip abduction exercise group compared to the control group	KOOS Pain Symptoms ADL Sports and Recreation Quality of Life	70 76 77 29 48	74 80 81 32 50	89 90 92 47 72	91 89 95 55 77
Wang et al	Sample size (*n* = 82) Strengthening Group (SG) (*n* = 41) Control Group (CG) (*n* = 41) Mean age of the SG group- 58.4(5.3) Mean age of the CG group- 59.2(6.1) Kellgran Lawrence (KL) grades of 2 and above were included on the study	Pain (VAS Scale) Physical Function (WOMAC) Strength was assessed by an Isokinetic Dynamometer Five Times Sit-to-Stand Test, stair ascent/descent task and Figure of 8 walk test.	Hip abductor strengthening and quadriceps exercises, when compared experimental group had superior outcomes in stair ascent/descent task, figure of 8 Walk test, and pain; but not in the Five Times Sit-to-Stand Test and Self-reported functional difficulties score	VAS WOMAC Fig. of 8 walk test FTSST Strength of hip Abduction Stair Ascent and Descent	5.70 ± 1.00 33.8 ± 9.9 10.92 ± 2.23 15.53 ± 3.95 1.16 ± 0.33 21.05 ± 3.96	5.50 ± 0.93 32.3 ± 7.5 10.47 ± 1.38 15.49 ± 2.60 1.16 ± 0.33 20.55 ± 2.59	3.12 ± 0.83 20.9 ± 6.0 8.58 ± 1.42 3.51 ± 3.77 1.31 ± 0.36 17.10 ± 3.23	3.54 ± 1.08 26.8 ± 5.9 75 ± 1.25 13.98 ± 2.69 1.18 ± 0.37 19.35 ± 2.83
Chaudhary Ashok	Sample size (*n* = 30) Strengthening Group (SG) (*n* = 15) Control Group (CG) (*n* = 15) Mean age of the SG group- 51.33(5.2326) Mean age of the CG group- 52(5.0142) Kellgren-Lawrence radiographic grade I, II and III were included in the study	Pain (VAS) Physical function (WOMAC)	Hip abductor muscle strengthening exercises showed overall improvement in pain and physical function and is a useful adjunct exercise therapy in treating patients with unilateral medial compartment knee osteoarthritis	VAS WOMAC	7 ± 1.690 66.66 ± 6.986	2 ± 1.463 27.66 ± 4.237	6.93 ± 1.387 67.13 ± 6.577	4.066 ± 1.907 37.46 ± 6.356
Elizabeth A. Sled	Sample Size (*n* = 40) Strengthening Group (SG) (*n* = 20) Control Group (CG) (*n* = 20) Mean age of the SG group- 62.98 (9.73) Mean age of the CG group- 62.98 (9.73) Kellgren-Lawrence radiographic grading was included.	Five-Times-Sit-To-Stand test WOMAC	Hip abductor strengthening did not reduce knee joint loading but did improve function and reduce pain in a group with medial knee OA.	Five-Times-Sit-To-Stand test WOMAC	15.2 (12.6–17.9) 19.60 (15.95–23.25)	12.5 (10.6–14.4) 18.15 (14.19–22.11)	10.1 (9.2–11.0) 1.2 (0.25–2.15)	9.3 (8.4–10.2) 1.24 (0.00–2.46)

*CI *Confidence interval, *CG* Control groups Strengthening group, *KL* Kellgren, and Lawrence, *KAM* Knee adduction moment, *MD* Mean difference, *NPRS* Numeric Pain Rating Scale, *OA* OA, *SF-36* 36-Item Short-Form Health Survey, *VAS* Visual analog scale, *KOOS* Knee OA Outcomes Survey, *WOMAC* Western Ontario and McMaster Universities OA Index, *1RM* One repetition Maximum. *CG* Control group, *SG* Strengthening group, *F* Frequency, *D* Duration, *1RM* One repetition Maximu

### Comparisons

Studies compared hip abductor strengthening with quadriceps strengthening, routine rehabilitation or no exercises**.**

#### Outcome

Assessment time points varied from 2 to 13 weeks. Three studies measured pain on the VAS and NPRS [26–28, 31]. 2 studies evaluated gait to measure knee adduction moment [27, 30]. Three studies measured physical function using the WOMAC [25, 26, 28, 30] while KOOS was also utilized by Yuenyongviwat et al. [29]. In addition, Wang et al. [28] analyzed the figure of 8 test, 6MWT, stair ascent and descent task. 2 studies analyzed five times sit to stand [28, 30]. One study investigated the health-related quality of life using the Short Form-36 questionnaire [26].

Meta – analysis was conducted for 3 of the 7 included studies. The homogenous outcomes analysed were VAS scores and the WOMAC score. For pain, three studies were analysed for the VAS scores, pre- and post-intervention. Heterogeneity [*I*^2^] was 9% (*p* < 0.0001). The mean difference was − 0.60 with [(95% confidence Interval) − 0.88 to − 0.33] for the intervention versus the control group. For functional outcome which was analysed using the WOMAC scores the heterogeneity [*I*^2^] was 17% (*p* < 0.0001). The mean difference was − 0.75 [(95% confidence interval − 1.05 to − 0.45] for the intervention against the control group (Figs. 2 and 3). According to the Cochrane Handbook 0% to 40%: might not be important; 30% to 60%: may represent moderate heterogeneity; 50% to 90%: may represent substantial heterogeneity; 75% to 100%: considerable heterogeneity [32]. In our study the heterogeneity is persistently below 40%, thereby being consistent with our result interpretation.

According to the findings of the included studies and their meta-analysis, hip abductor strength training reduces pain and improves functional outcomes in people with knee OA. The above values are displayed in a small confidence interval range, indicating the analyses' validity and sensitivity, as well as the significant influence. Furthermore, the random-effects model used provided accurate results by using sample size and standard error. The meta-analysis likewise comes up with a positive conclusion for hip abductor strength training. Even though the key outcome measures of VAS and WOMAC scores were homogeneous the analysed studies differed in the mode and duration of intervention and hence a meta-analysis was conducted using the random-effects model.

### Effects of intervention

Table 4 summarizes the conclusions of the investigations. Bennell et al. [27] conducted a study in which they evaluated hip adductor and abductor strengthening efficacy to no therapy. Pain, function, and hip strength all improved considerably after 12 weeks of home-based training, but there was no significant difference between groups in knee adduction moment change. [mean difference (95 percent confidence interval (CI)) 0.134 (- 0.069 to 0.337) Nm/BW * HT percent]. The pain, physical performance, and muscular strength assessments all improved significantly in the strengthening group.(*p *< 0.05).

Another study conducted by Sled et al. [30] concluded that, hip abductor strength of the OA group improved significantly after the treatment, but not the knee adduction moment. When compared to the control group, the OA group's functional performance on the sit-to-stand test improved. Jorge et al. [26] found improvements in WOMAC and knee pain [Exercise group—pain (from 7.01.3 to 4.33.1 in the Exercise group and from 7.01.2 to 6.61.5 in the Control group- *p* < 0.001)] when compared to no intervention and several aspects of quality of life, muscle strength, walking distance and velocity following 12 weeks of training.

Singh et al. [26] revealed that when hip abductor muscle strengthening was compared to traditional exercises the results improved on the WOMAC scores and the 6MWT. Yuenyongviwat et al. [29], statistical analysis revealed both groups had significantly improved KOOS pain at 10 weeks. (Hip abductor exercise group + 18.68 (95% CI, 11.8–25.6, *p* < 0.01). The other subscales also showed improvement at 10 weeks (p < 0.01). When compared to the knee exercise group, the effects of exercise on pain management and numerous subscales were observed statistically significant in the hip abduction exercise group.

According to Wang et al. [28], In the stair descent and ascent task, the five-time sit to stand test, the Figure of 8 walk test, and the functional outcome score, the intervention group outperformed the control group. At the sixth week, there were significant variations in WOMAC and VAS scores between groups. (*p* < 0.05).

Study conducted by Chaudry A [31], concluded that the intervention group improved significantly on pain and functional outcomes after 6 weeks of intervention.

Seven studies assessed the effectiveness of hip abductor strengthening in comparison to routine exercises or no exercise in the control group in subjects with knee osteoarthritis.. All the included studies showed statistically significant differences for the assessed outcomes between the study groups and pre-post intervention. There was a clinically significant difference between the pain scores, the functional outcome scores and the hip abductor strength. However, there was no statistically significant difference in the knee joint loading post hip abductor strengthening in the intervention group.

### Adverse events

Following exercise intervention, two studies concluded that the subjects in the intervention group complained of exacerbated knee pain along with back and hip discomfort [26, 27]. In another study [28] however, muscular discomfort was predominant in both the control and experimental groups.

## Discussion

The aim of the present review was to identify the effectiveness of hip abductor strengthening in individuals diagnosed with knee osteoarthritis. This systematic review included findings from 7 RCT’s that reviewed the current evidence on hip abductor strengthening in knee OA. According to the evidence gathered, hip abductor strengthening effectively relieved knee discomfort by lowering pain scores, improving self-reported functional outcomes, physical performance and providing an overall sense of well-being. International guidelines and several other studies recommend therapeutic intervention or exercises as a crucial component of conservative management of knee OA [33, 34]. Weak knee extensors have been identified as one of the contributors to the onset and progression of OA but it is likely that other than knee extensors, weakness in various other muscle groups could also contribute to reduced function in subjects with knee OA [33, 35, 36]. This review examined articles that discussed the impact of hip abductor strength training on disease progression and medial joint loading in knee OA.

Hip abductors are important for supporting and stabilizing the trunk and assisting in limb placement control during functional tasks. Weakness of hip abductor muscles are known to compromise mediolateral stability at the pelvis, leading to abnormal gait mechanics. During walking, torque generation of hip abductors in the stance phase stabilizes the pelvis as the position of the pelvis can alter the body’s centre of mass thereby altering knee joint loading. Weak hip abductors cause pelvic drop towards the contralateral swing leg thus shifting the body’s Centre of Mass away from the centre of the knee joint [37]. This in turn increases adduction moment in hip leading to rapid progression of arthritic changes in the knee [17–21, 25].

Hip abductor weakness is associated with functional decline as it impacts the force generation [24] which in turn alters the joint loading and structural progression during weight-bearing activities. There is also reduced medial tibiofemoral disease progression due to pelvic control in the frontal plane which in turn prevents the shift of line of gravity from the stance knee and reduces adduction moment. Studies suggest that hip abductor strength training may reduce hip adduction moment causing a decrease in medial compartment loading thereby decreasing pain and disease progression by improving physical functional scores, peak hip adduction angle and reduction in knee joint loading [23].

Hip abduction activation is required to maintain postural stability and balance during walking and transfers [23, 38]. Hip abductors are thought to play a role in dynamic postural control, particularly lateral stability control, and strengthening these muscle groups has resulted in improved hip motor control during functional activities [39]. Hip abductors comprise of the gluteus medius as the prime mover and rectus femoris, gluteus minimus, tensor fascia latae, sartorius as assistant movers [40]. Exercise programs opted should be based on the physical fitness and preference of the patient with knee osteoarthritis. A targeted exercise regimen of the hip abductors might reduce the loading on the knee joint's medial compartment which could significantly improve knee symptoms [41]. Exercise programs designed for a duration of 3 to 5 times per week, for a period of 6 to 9 weeks are known to result in favourable outcomes [42].

Conservative management in knee osteoarthritis mainly revolves around reducing the mechanical load on the joint. This can be done by reinforcing the lower extremity muscle strength, especially the quadriceps muscle which not only impacts the onset and progression of disease but also plays a major role in activity limitation in subjects with knee osteoarthritis. Biomechanical factors play a major role in the onset and progression of knee osteoarthritis [43–46]. Strengthening of quadriceps not only assists in the reduction of knee joint load but also protects the articular cartilage [7, 8, 14]. Hip abductor muscles significantly affect the knee joint loading which is a modifiable factor contributing to disease progression. The strength training parameters in the included trials were constant with the guidelines for strength exercises in subjects with knee osteoarthritis [47]. The intensity and dosage of exercise in the included articles varied from 50 to 80% of 1 repetition maximum or 10 repetition maximum, performed 3 to 5 times a week with an intensity of 8 – 20 repetitions for 2 to 3 sets. VAS and NPRS, both of which are deemed reliable and valid were used to assess improvements in knee pain Strengthening the hip abductors was done either in standing or side-lying and using free weights or elastic TheraBand’s (Table 3). The exercises significantly improved the strength of the hip abductors, and an uptrend was seen with regards to the functional outcome scores [25–29].

In accordance with the OARSI recommendations, which suggests five physical tests to assess the functional capacities of persons with knee osteoarthritis [47] and three tests to access physical performance: 30-s chair stand test, Timed up and Go Test, 6-min walk test, 40-m rapid walking test, 9-step stair climb test and 6-min walk test, step test and stair climb test. The included studies used performance-based measures such as short- and long-distance activity (6MWT) and stair negotiation activity (step test). Three-dimensional gait analysis and motion measuring devices were used to measure the biomechanical metrics of knee loads and dynamic alignment [25–29].

For subjects with knee OA, rehabilitation strategies along with adjunct therapies like taping and use of knee and patellar bracing can be implemented. In people with knee OA, donning a soft knee brace has been proven to minimise self-reported knee instability. Braces are designed to promote hamstring activation and variable degree of knee extension moment are known to promote pain-free weightbearing activities by reducing the moment of compressive forces produced by the quadriceps [48]. Foot orthoses also have the possibility to be an effectual treatment for knee OA. Footwear which are contoured and prefabricated produce an immediate relief in pain in knee OA patients during functional activities and produce an ease in task performance. Heeled footwear may reduce the efficiency of a lateral wedged insole. The best way to use a lateral wedged insole for knee OA is in conjunction with socks or flat footwear without heels [49].

Exercises are recommended with a focus on lower limb strengthening. Hip abductor muscle strengthening, either alone or in combination with lower extremity exercises, improved symptoms without having a substantial impact on medial compartment knee loading measurements. Because OA causes long-term disability, treatment delivery strategies that meet the ongoing need for therapy are imperative. Home-based rehabilitation though ensures long-term delivery of treatment, adherence to exercises is a disadvantage of unsupervised training.

### Clinical implications

Both low- and high-resistance exercise programmes improved knee pain and function. The biomechanical parameters of knee joint loading were unaffected by either the low or high resistance regimens [27, 30]. As a result, the intensity of hip abductor strengthening exercises must be chosen based on the preferences and general conditioning of individuals with knee OA. The recommended quantity for exercise frequency, according to the included studies, is 3 to 5 times per week. Exercise therapy lasted 6 weeks to 3 months and produced significant outcomes. As a result, hip abductor strengthening is useful for short to moderate amount of time. The long-term implications should be investigated. The collective evidence of this review will provide clinicians with an insight into choosing the proper therapeutic approach in treating patients diagnosed with knee OA.

### Limitations and future scope

There are certain limitations to this systematic review. The included studies were conducted on a small sample size, hence extrapolating the results to a large population is difficult. The included studies failed to ascertain the role of other hip musculature and their impact on disease progression. The physical activity level of the subjects was not considered in any of the included research. One of the included studies only involved women, making it impossible to extend the findings to other genders. Further research is needed, particularly concerning the intermediate and long-term effects of hip- abductor focused resistance and neuromuscular functional training in knee OA. In addition to high-intensity resisted quadriceps strengthening, future research should look into the benefits of high-intensity resisted hip abductor strength training for patient-reported outcomes. More research is needed to assess the relative effectiveness of open and closed kinematic chain hip exercises in subjects with knee OA.

## Conclusion

Knee OA is a disabling condition as it affects individuals both functionally and psychologically. Muscle weakness is known to be one of the major contributing factors for disease progression [50]. Evidence suggests that weakness of the hip abductors reduces the propulsion or explosive force in weight-bearing activities, which in turn stresses the medial tibiofemoral joint and leads to disease progression [22, 23]. The current review and meta-analysis identified a positive relationship between hip abductor strengthening and knee osteoarthritis. Strengthening the hip abductors resulted in an improvement in the functional scores and a relative reduction in the pain intensity. These positive findings suggest that hip abductor strengthening can be used as an effective exercise regime in subjects with knee OA, but further work is required to explore whether these benefits on the assessed functional outcomes are maintained for a long period of time. Thus, the findings of this review have provided us with an understanding of the influence, effect and importance of hip abductor strengthening in patients diagnosed with knee osteoarthritis.

## Supplementary Information

**Additional file 1.**

## Data Availability

The data used to support the findings of this study are available in the text and can be procured from the corresponding author upon request.

## Abbreviations

KOOS: Knee Injury and Osteoarthritis Outcome Score
NPRS: Numerical Pain Rating Scale
OA: Osteoarthritis
RCT: Randomized controlled trials
SF-36: Short Form- 36
6MWT: Six-minute walk test
VAS: Visual Analogue Scale
WOMAC: Western Ontario and McMaster Universities

## References

[1] Kim I, Kim HA, Seo Y-I, Song YW, Hunter DJ, Jeong JY (2010). Tibiofemoral osteoarthritis affects quality of life and function in elderly Koreans, with women more adversely affected than men. BMC Musculoskelet Disord.

[2] Ledingham J, Regan M, Jones A, Doherty M (1993). Radiographic patterns and associations of osteoarthritis of the knee in patients referred to hospital. Ann Rheum Dis.

[3] Iorio R, Healy WL (2003). Unicompartmental arthritis of the knee. J Bone Joint Surg Am.

[4] Cui A, Li H, Wang D, Zhong J, Chen Y, Lu H (2020). Global, regional prevalence, incidence and risk factors of knee osteoarthritis in population-based studies. EClinicalMedicine.

[5] Pal CP, Singh P, Chaturvedi S, Pruthi KK, Vij A (2016). Epidemiology of knee osteoarthritis in India and related factors. Indian J Orthop.

[6] McDonough CM, Jette AM (2010). The contribution of osteoarthritis to functional limitations and disability. Clin Geriatr Med.

[7] Farrokhi S, Voycheck CA, Tashman S, Fitzgerald GK (2013). A biomechanical perspective on physical therapy management of knee osteoarthritis. J Orthop Sports Phys Ther.

[8] Thorp LE, Sumner DR, Block JA, Moisio KC, Shott S, Wimmer MA (2006). Knee joint loading differs in individuals with mild compared with moderate medial knee osteoarthritis. Arthritis Rheum.

[9] Miyazaki T, Wada M, Kawahara H, Sato M, Baba H, Shimada S (2002). Dynamic load at baseline can predict radiographic disease progression in medial compartment knee osteoarthritis. Ann Rheum Dis.

[10] Thorp LE, Sumner DR, Wimmer MA, Block JA (2007). Relationship between pain and medial knee joint loading in mild radiographic knee osteoarthritis. Arthritis Care Res.

[11] Pietrosimone B, Thomas AC, Saliba SA, Ingersoll CD (2014). Association Between Quadriceps Strength And Self-Reported Physical Activity In People With Knee Osteoarthritis. Int J Sports Phys Ther.

[12] Bennell KL, Wrigley TV, Hunt MA, Lim BW, Hinman RS (2013). Update on the Role of Muscle in the Genesis and Management of Knee Osteoarthritis. Rheum Dis Clin North Am..

[13] Van Der Esch M, Holla JF, Van Der Leeden M, Knol DL, Lems WF, Roorda LD (2014). Decrease of muscle strength is associated with increase of activity limitations in early knee osteoarthritis: 3-year results from the cohort hip and cohort knee study. Arch Phys Med Rehabil.

[14] Tuna S, Balcı N, Özçakar L (2016). The relationship between femoral cartilage thickness and muscle strength in knee osteoarthritis. Clin Rheumatol.

[15] Hinman RS, Wrigley TV, Metcalf BR, Hunter DJ, Campbell P, Paterson K (2014). Unloading shoes for osteoarthritis of the knee: protocol for the SHARK randomised controlled trial. BMC Musculoskelet Disord.

[16] Schipplein OD, Andriacchi TP (1991). Interaction between active and passive knee stabilizers during level walking. J Orthop Res.

[17] Rutherford DJ, Hubley-Kozey C, Stanish W (2014). Hip abductor function in individuals with medial knee osteoarthritis: Implications for medial compartment loading during gait. Clin Biomech.

[18] Costa RA, de Oliveira LM, Watanabe SH, Jones A, Natour J (2010). Isokinetic assessment of the hip muscles in patients with osteoarthritis of the knee. Clinics.

[19] Lun V, Marsh A, Bray R, Lindsay D, Wiley P (2015). Efficacy of Hip Strengthening Exercises Compared With Leg Strengthening Exercises on Knee Pain, Function, and Quality of Life in Patients With Knee Osteoarthritis. Clin J Sport Med.

[20] Tevald MA, Murray A, Luc BA, Lai K, Sohn D, Pietrosimone B (2016). Hip abductor strength in people with knee osteoarthritis: A cross-sectional study of reliability and association with function. Knee.

[21] Hinman RS, Hunt MA, Creaby MW, Wrigley TV, McManus FJ, Bennell KL (2010). Hip muscle weakness in individuals with medial knee osteoarthritis. Arthritis Care Res.

[22] Mündermann A, Dyrby CO, Andriacchi TP (2005). Secondary gait changes in patients with medial compartment knee osteoarthritis: Increased load at the ankle, knee, and hip during walking. Arthritis Rheum.

[23] Chang A, Hayes K, Dunlop D, Song J, Hurwitz D, Cahue S (2005). Hip abduction moment and protection against medial tibiofemoral osteoarthritis progression. Arthritis Rheum.

[24] Chang AH, Chmiel JS, Almagor O, Hayes KW, Guermazi A, Prasad PV (2019). Hip muscle strength and protection against structural worsening and poor function and disability outcomes in knee osteoarthritis. Osteoarthr Cartil.

[25] Singh S, Pattnaik M, Mohanty P, Ganesh GS (2016). Effectiveness of hip abductor strengthening on health status, strength, endurance and six minute walk test in participants with medial compartment symptomatic knee osteoarthritis. J Back Musculoskelet Rehabil.

[26] Jorge RTB, de Souza MC, Chiari A, Jones A, da RC Fernandes A, Lombardi Júnior I (2015). Progressive resistance exercise in women with osteoarthritis of the knee: a randomized controlled trial. Clin Rehabil.

[27] Bennell KL, Hunt MA, Wrigley TV, Hunter DJ, McManus FJ, Hodges PW (2010). Hip strengthening reduces symptoms but not knee load in people with medial knee osteoarthritis and varus malalignment: A randomised controlled trial. Osteoarthr Cartil.

[28] Wang J, Xie Y, Wang L, Lei L, Liao P, Wang SQ (2020). Hip abductor strength–based exercise therapy in treating women with moderate-to-severe knee osteoarthritis: a randomized controlled trial. Clin Rehabil.

[29] Yuenyongviwat V, Duangmanee S, Iamthanaporn K, Tuntarattanapong P, Hongnaparak T, Yuenyongviwat V DSIKTP (2020). Effect of hip abductor strengthening exercises in knee osteoarthritis: a randomized controlled trial. BMC Musculoskelet Disord.

[30] Sled EA, Khoja L, Deluzio KJ, Olney SJ, Culham EG (2010). Effect of a home program of hip abductor exercises on knee joint loading, strength, function, and pain in people with knee osteoarthritis: A clinical trial. Phys Ther.

[31] Chaudhary A (2012). Effects of Hip Abductor Muscle Strengthening Exercises in Patients with Osteoarthritic Knee Joints. Indian J Physiother Occup Ther Int J.

[32] Higgins J, Thomas J. Chapter 10: Cochrane handbook for systematic reviews of interventions. 2022.

[33] Juhl C, Christensen R, Roos EM, Zhang W, Lund H (2014). Impact of exercise type and dose on pain and disability in knee osteoarthritis: A systematic review and meta-regression analysis of randomized controlled trials. Arthritis Rheumatol.

[34] M F, S M, AR H, M V der E, M S, KL B. Exercise for osteoarthritis of the knee: a Cochrane systematic review. Br J Sports Med. 2015;49(24):1554–7. 10.1136/bjsports-2015-095424 26405113

[35] McAlindon TE, Cooper C, Kirwan JR, Dieppe PA (1993). Determinants of disability in osteoarthritis of the knee. Ann Rheum Dis.

[36] Berger MJ, McKenzie CA, Chess DG, Goela A, Doherty TJ (2012). Quadriceps neuromuscular function and self-reported functional ability in knee osteoarthritis. J Appl Physiol.

[37] Moisio K, Colbert C, Almagor O, Chmiel J, Chang A, Zhang J (2011). Sagittal plane hip motion during gait and function and disability in knee osteoarthritis. Osteoarthr Cartil.

[38] Deasy M, Leahy E, Semciw AI (2016). Hip Strength Deficits in People With Symptomatic Knee Osteoarthritis: A Systematic Review With Meta-analysis. J Orthop Sport Phys Ther.

[39] Be M, We M (2005). Change-in-support balance reactions in older persons: an emerging research area of clinical importance. Neurol Clin.

[40] Anderson LC, Blake DJ (1994). The anatomy and biomechanics of the hip joint. Garden FH, editor.. J Back Musculoskelet Rehabil.

[41] Thorp LE, Wimmer MA, Foucher KC, Sumner DR, Shakoor N, Block JA (2010). The biomechanical effects of focused muscle training on medial knee loads in OA of the knee: A pilot, proof of concept study. J Musculoskelet Neuronal Interact.

[42] Raghava Neelapala YV, Bhagat M, Shah P (2020). Hip Muscle Strengthening for Knee Osteoarthritis: A Systematic Review of Literature. J Geriatr Phys Ther.

[43] Astephen JL, Deluzio KJ, Caldwell GE, Dunbar MJ (2008). Biomechanical changes at the hip, knee, and ankle joints during gait are associated with knee osteoarthritis severity. J Orthop Res.

[44] Varady NH, Grodzinsky AJ (2016). Osteoarthritis year in review 2015: Mechanics. Osteoarthritis Cartilage..

[45] Saxby DJ, Lloyd DG (2017). Osteoarthritis year in review 2016: mechanics. Osteoarthritis Cartilage..

[46] Hunt MA, Wrigley TV, Hinman RS, Bennell KL (2010). Individuals with severe knee osteoarthritis (OA) exhibit altered proximal walking mechanics compared with individuals with less severe OA and those without knee pain. Arthritis Care Res.

[47] Dobson F, Hinman RS, Roos EM, Abbott JH, Stratford P, Davis AM (2013). OARSI recommended performance-based tests to assess physical function in people diagnosed with hip or knee osteoarthritis. Osteoarthr Cartil.

[48] Cudejko T, Van Der Esch M, Schrijvers J, Richards R, Van Den Noort JC, Wrigley T (2018). The immediate effect of a soft knee brace on dynamic knee instability in persons with knee osteoarthritis. Rheumatology (Oxford).

[49] Toda Y, Tsukimura N (2008). Influence of concomitant heeled footwear when wearing a lateral wedged insole for medial compartment osteoarthritis of the knee. Osteoarthr Cartil.

[50] Alnahdi AH, Zeni JA, Snyder-Mackler L (2012). Muscle Impairments in Patients With Knee Osteoarthritis. Sports Health.

